# The genome sequence of the great crested newt, *Triturus cristatus *(Laurenti, 1768)

**DOI:** 10.12688/wellcomeopenres.24257.1

**Published:** 2025-05-23

**Authors:** Jeffrey W. Streicher, Stephanie Holt, Natural History Museum Genome Acquisition Lab, Wellcome Sanger Institute Tree of Life Management, Samples and Laboratory team, Wellcome Sanger Institute Scientific Operations: Sequencing Operations, Wellcome Sanger Institute Tree of Life Core Informatics team, Tree of Life Core Informatics collective, Darwin Tree of Life Consortium

**Affiliations:** 1 Natural History Museum, London, England, UK; 2 University of Oxford, Oxford, England, UK

**Keywords:** Triturus cristatus, (Great or Northern) Crested Newt, genome sequence, chromosomal, Caudata

## Abstract

We present a genome assembly from a female specimen of *Triturus cristatus* (great crested newt; Chordata; Amphibia; Caudata; Salamandridae). The genome sequence has a total length of 22,324.62 megabases. Most of the assembly (98.78%) is scaffolded into 12 chromosomal pseudomolecules. The mitochondrial genome has also been assembled, with a length of 16.54 kilobases.

## Species taxonomy

Eukaryota; Opisthokonta; Metazoa; Eumetazoa; Bilateria; Deuterostomia; Chordata; Craniata; Vertebrata; Gnathostomata; Teleostomi; Euteleostomi; Sarcopterygii; Dipnotetrapodomorpha; Tetrapoda; Amphibia; Batrachia; Caudata; Salamandroidea; Salamandridae; Pleurodelinae; *Triturus*; *Triturus cristatus* (Laurenti, 1768) (NCBI:txid8323)

## Background

The great crested newt, *Triturus cristatus*, is widely distributed throughout northern Eurasia. It occurs in mixed and deciduous forests of Austria, Belarus, Belgium, Czech Republic, Denmark, Estonia, Finland, France, Germany, Latvia, Liechtenstein, Lithuania, Luxembourg, Moldova, the Netherlands, Norway, Poland, Romania, the Russian Federation, Serbia, Slovakia, Sweden, Switzerland, Ukraine, and the United Kingdom ( AmphibiaWeb, 2021). Also known as northern crested newt or great warty newt, the striking dorsal crest observed in breeding adult males has been used to study the evolutionary origin of phenotypic plasticity and novel traits ( Wiens *et al.*, 2011). Populations of *T. cristatus* in Great Britain are widely distributed in England and found in some parts of Wales and Scotland ( Arnold, 1995). The species is protected by law in the United Kingdom with robust monitoring programmes in place ( Buxton & Griffiths, 2022; Griffiths *et al.*, 2010). Known to be susceptible to the fungal pathogen *Batrachochytrium salamdrividans*, *T. cristatus* is a Biodiversity Action Plan priority species, listed on Appendix II of the Bern Convention and on Annexes II and IV of the EU Natural Habitats Directive in Europe ( Cunningham *et al.*, 2019).

**Figure 1.**
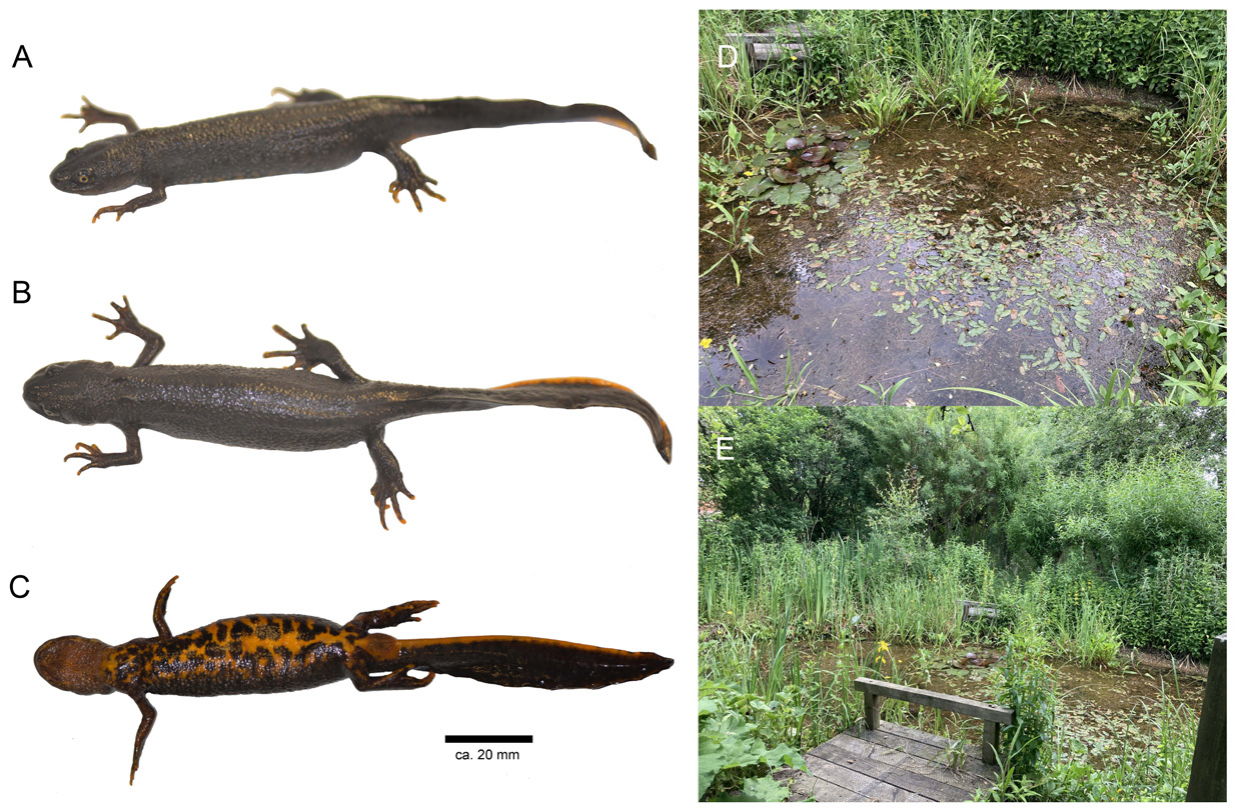
Female voucher specimen of *Triturus cristatus* (NHMUK 2022.7580, Field ID, JWS 916; Snout-vent length 82.6 mm) from which the genome was sequenced in lateral ( **A**), dorsal ( **B**), and ventral ( **C**) views. The individual was collected from Gilbert White’s House and Gardens in Selbourne, England. Two views of the garden pond where the specimen was collected via dip netting ( **D**– **E**).

The genome was sequenced from one female *T. cristatus* ( Figure 1A, B, C) collected by net from a garden pond at Gilbert White’s House and Gardens in Selbourne, Hampshire, England – a museum and site of historical significance in British natural history. The museum, located at The Wakes – White’s home until his death in 1793 – commemorates his life and work as a pioneering naturalist and educator, and hosts exhibitions on other figures in exploration and natural history. White was a pioneering English naturalist who is often credited as England’s first ecologist, based on his book, *The Natural History and Antiquities of Selborne* ( White, 1789), journals, and earlier publications in the *Philosophical Transactions of the Royal Society*. His work marked a step-change in observational natural history and influenced much contemporary work such as Thomas Pennant’s groundbreaking *British Zoology* (1778), as well as many who followed him, including Charles Darwin and Sir David Attenborough. In *The Natural History and Antiquities of Selborne*, he discusses English newts in relation to the developing knowledge of complex and diverse amphibian lifecycles: ‘I used to take it for granted that the *salamandra aquatica* [the newt] was hatched, lived, and died in the water. But John Ellis, Esq., F.R.S. (the coralline Ellis) asserts, in a letter to the Royal Society, dated June the 5th, 1766, in his account of the mud inguana [sic], an amphibious bipes [a reference to the obligate aquatic salamander *Siren lacertina*, which like the squamate genus *Bipes* possesses only forelimbs], from South Carolina, that the water-eft, or newt, is only the larva of the land-eft, as tadpoles are of frogs’ ( White, 1789). This early recognition of different life histories in caudatan amphibians, where some remain completely aquatic as adults, retaining larval features like external gills (e.g. *Siren lacertina*), while others undergo metamorphosis to become terrestrial (or semi-terrestrial) adults. (e.g. *Triturus cristatus*), marked an important advance in understanding the ecological diversity of amphibians.

The great crested newt and its close relative *T. marmoratus* have an atypical heteromorphy in their genomes; a chromosome 1 syndrome ( Macgregor & Horner, 1980; Wielstra, 2020). This syndrome leads to a so-called ‘balanced lethal system’ which results in mortality of 50% of their offspring ( Wielstra, 2020). The 50% mortality in *T. cristatus* is explained by each variant of chromosome 1 containing a lethal recessive allele. In offspring that are homozygous for either of the chromosome morphs, development ceases halfway through embryogenesis, whereas development completes in heterozygous individuals ( Fahrbach & Gerlach, 2018). The evolutionary origins of (seemingly wasteful) balanced lethal systems remain enigmatic but could be related to ancestral sex chromosomes ( Grossen *et al.*, 2012) or collapsed supergenes ( Schwander *et al.*, 2014). In our assembly, we see evidence of chromosome 1 being heteromorphic with one arm of chromosome 1 having half the coverage expected.

Like other salamanders and newts (Amphibia: Caudata), *T. cristatus* has an extremely large genome ( Liedtke *et al.*, 2018), with previous c-value estimates as large as 27.77 pg ( Litvinchuk *et al.*, 2007). All cytogenetics studies of *Triturus* species (including *T. cristatus*) have reported a diploid 2 *n* = 24 ( Baldwin & Macgregor, 1985), which matches the 12 chromosomes resulting from our assembly. Due to their extremely large size, five chromosomes were split into two pseudomolecules each, resulting in 17 pseudomolecules total. The estimated size of the genome (22 Gb) is larger than a previously sequenced salamandrid genome from *Pleurodeles walti* (20 Gb, Brown *et al.,* 2025), but smaller than the genome sequenced from the axolotl, *Ambystoma mexicanum* (32 Gb, Nowoshilow *et al.*, 2018). The chromosome-level whole genome sequence for *T. cristatus* presented here was produced as part of the Darwin Tree of Life Project, Sanger Tree of life Project and Vertebrate Tree of Life Project.

## Genome sequence report

### Sequencing data

The genome of a specimen of *Triturus cristatus* ( Figure 1) was sequenced using Pacific Biosciences single-molecule HiFi long reads, generating 809.03 Gb from 65.40 million reads, which were used to assemble the genome. GenomeScope analysis estimated the haploid genome size at 21,641.98 Mb (22 Gb), with a heterozygosity of 0.28% and repeat content of 48.53%. These estimates guided expectations for the assembly. Based on the estimated genome size, the sequencing data provided approximately 36 coverage. Hi-C sequencing produced 920.77 Gb from 6,097.79 million reads, and was used to scaffold the assembly. RNA sequencing data were also generated and are available in public sequence repositories. Table 1 summarises the specimen and sequencing details.

**Table 1.** Specimen and sequencing data for *Triturus cristatus*.

Platform	PacBio HiFi	Hi-C	RNA-seq
**ToLID**	aTriCri1	aTriCri1	aTriCri1
**Specimen ID**	NHMUK014561658	NHMUK014561658	NHMUK014561658
**BioSample (source** **individual)**	SAMEA112468129	SAMEA112468129	SAMEA112468129
**BioSample (tissue)**	SAMEA112468181	SAMEA112468178	SAMEA112468179
**Tissue**	liver	muscle	ovary
**Sequencing** **platform and model**	Revio	Illumina NovaSeq 6000	Illumina NovaSeq X
**Run accessions**	ERR13033468; ERR13033469; ERR13033470; ERR13033471; ERR13033476; ERR13033467; ERR13033472; ERR13033475; ERR13033473; ERR13033474	ERR13063105	ERR13493948
**Read count total**	65.40 million	6 097.79 million	112.22 million
**Base count total**	809.03 Gb	920.77 Gb	16.95 Gb

### Assembly statistics

The primary haplotype was assembled, and contigs corresponding to an alternate haplotype were also deposited in INSDC databases. The assembly was improved by manual curation, which corrected 324 misjoins or missing joins. These interventions reduced the total assembly length by 5.49%, decreased the scaffold count by 12.6%, and also decreased the scaffold N50 by 32.78%. The final assembly has a total length of 22,324.62 Mb in 1,303 scaffolds, with 4,698 gaps, and a scaffold N50 of 1248.77 Mb ( Table 2).

**Table 2.** Genome assembly data for *Triturus cristatus*.

Genome assembly		
Assembly name	aTriCri1.1	
Assembly accession	GCA_964204655.1	
*Alternate haplotype accession*	*GCA_964204665.1*	
Assembly level for primary assembly	chromosome	
Span (Mb)	22,324.62	
Number of contigs	6,001	
Number of scaffolds	1,303	
Longest scaffold (Mb)	1950.67	
Assembly metric	Measure	*Benchmark*
Contig N50 length	8.5 Mb	*≥ 1 Mb*
Scaffold N50 length	1,248.77 Mb	*= chromosome N50*
Consensus quality (QV)	Primary: 65.4; alternate: 60.6; combined: 62.4	*≥ 40*
*k*-mer completeness	Primary: 94.04%; alternate: 45.11%; combined: 99.07%	*≥ 95%*
BUSCO *	C:92.7%[S:88.8%,D:3.8%], F:2.3%,M:5.0%,n:5,310	*S > 90%; D < 5%*
Percentage of assembly mapped to chromosomes	98.78%	*≥ 90%*
Sex chromosomes	Not identified	*localised homologous pairs*
Organelles	Mitochondrial genome: 16.54 kb	*complete single alleles*

* BUSCO scores based on the tetrapoda_odb10 BUSCO set using version 5.7.1. C = complete [S = single copy, D = duplicated], F = fragmented, M = missing, n = number of orthologues in comparison.

**Figure 2.**
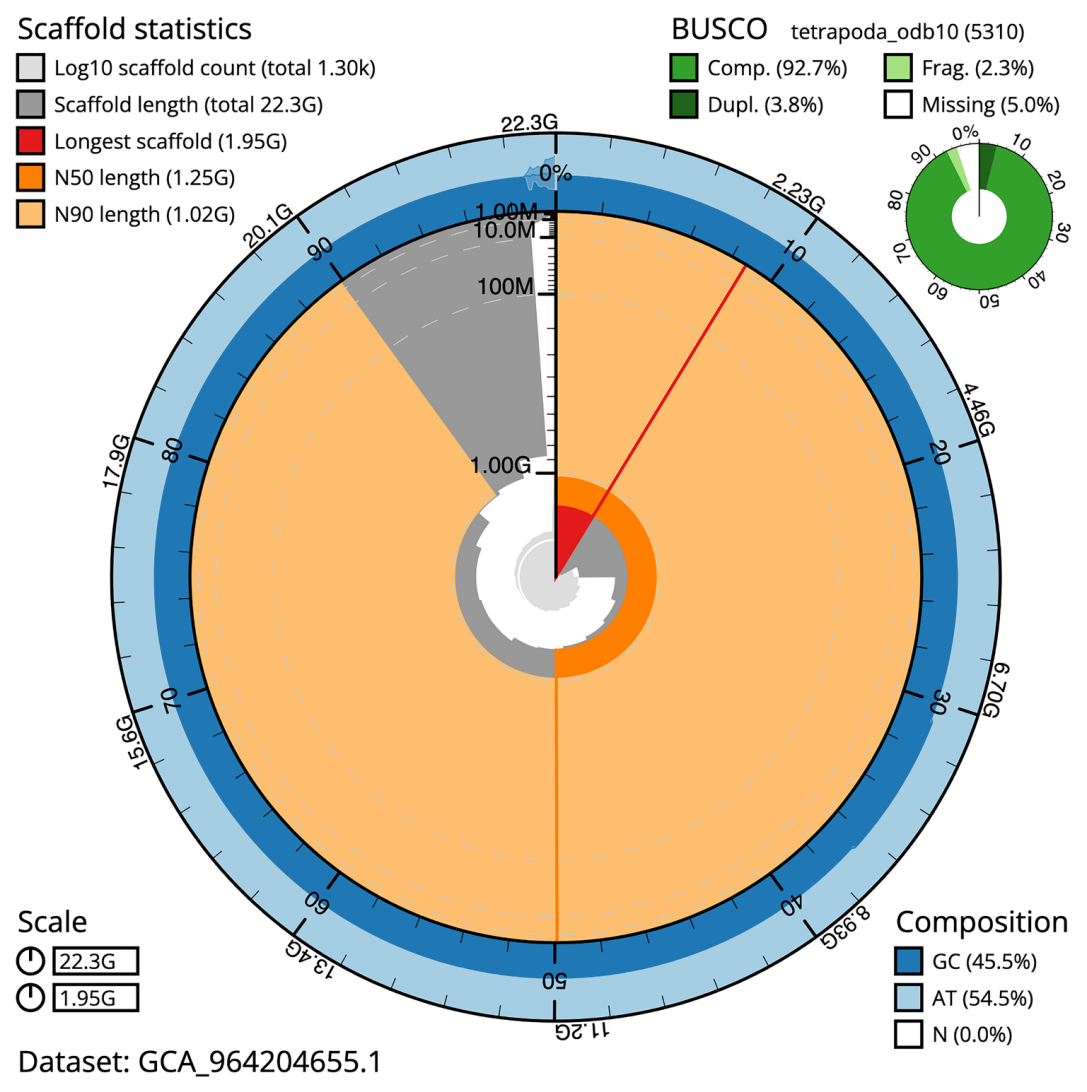
Genome assembly of *Triturus cristatus*, aTriCri1.1: metrics. The BlobToolKit snail plot provides an overview of assembly metrics and BUSCO gene completeness. The circumference represents the length of the whole genome sequence, and the main plot is divided into 1,000 bins around the circumference. The outermost blue tracks display the distribution of GC, AT, and N percentages across the bins. Scaffolds are arranged clockwise from longest to shortest and are depicted in dark grey. The longest scaffold is indicated by the red arc, and the deeper orange and pale orange arcs represent the N50 and N90 lengths. A light grey spiral at the centre shows the cumulative scaffold count on a logarithmic scale. A summary of complete, fragmented, duplicated, and missing BUSCO genes in the tetrapoda_odb10 set is presented at the top right. An interactive version of this figure is available at https://blobtoolkit.genomehubs.org/view/GCA_964204655.1/dataset/GCA_964204655.1/snail.

**Figure 3.**
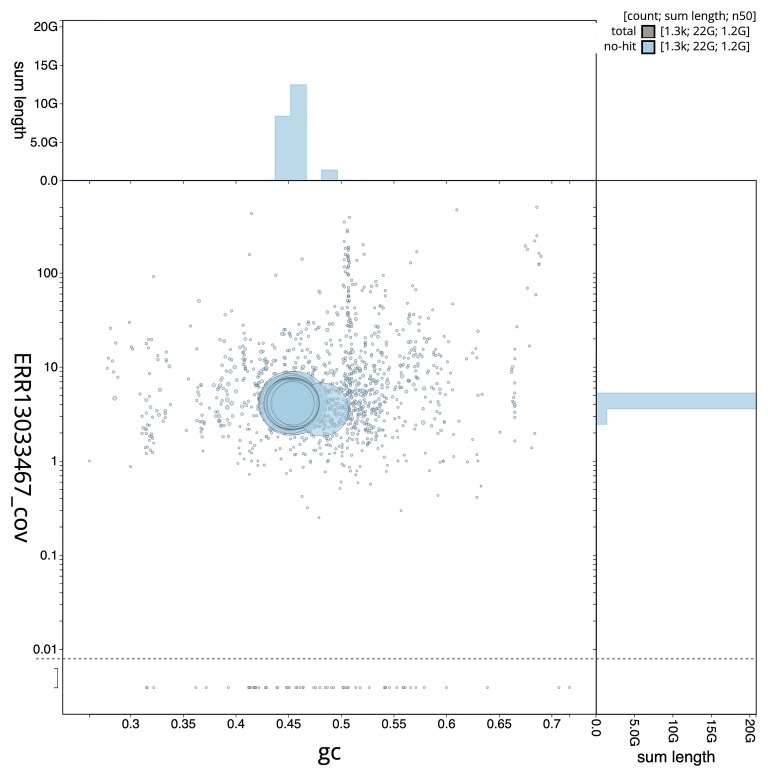
Genome assembly of *Triturus cristatus*, aTriCri1.1: BlobToolKit GC-coverage plot. Blob plot showing sequence coverage (vertical axis) and GC content (horizontal axis). The circles represent scaffolds, with the size proportional to scaffold length and the colour representing phylum membership. The histograms along the axes display the total length of sequences distributed across different levels of coverage and GC content. An interactive version of this figure is available at https://blobtoolkit.genomehubs.org/view/GCA_964204655.1/dataset/GCA_964204655.1/blob.

**Figure 4.**
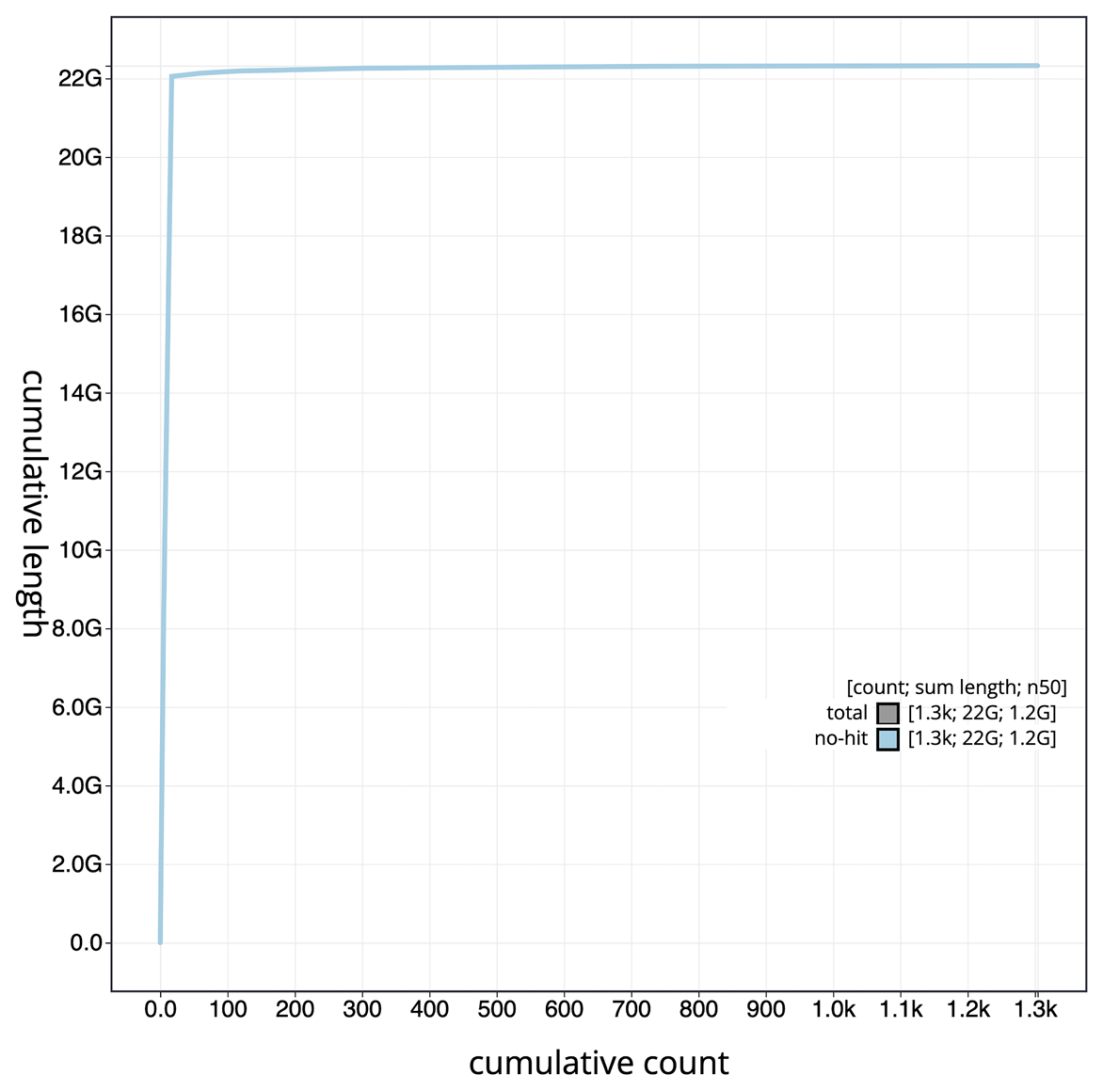
Genome assembly of *Triturus cristatus,* aTriCri1.1: BlobToolKit cumulative sequence plot. The grey line shows cumulative length for all scaffolds. Coloured lines show cumulative lengths of scaffolds assigned to each phylum using the buscogenes taxrule. An interactive version of this figure is available at https://blobtoolkit.genomehubs.org/view/GCA_964204655.1/dataset/GCA_964204655.1/cumulative.

The snail plot in Figure 2 provides a summary of the assembly statistics, indicating the distribution of scaffold lengths and other assembly metrics. Figure 3 shows the distribution of scaffolds by GC proportion and coverage. Figure 4 presents a cumulative assembly plot, with separate curves representing different scaffold subsets assigned to various phyla, illustrating the completeness of the assembly.

**Figure 5.**
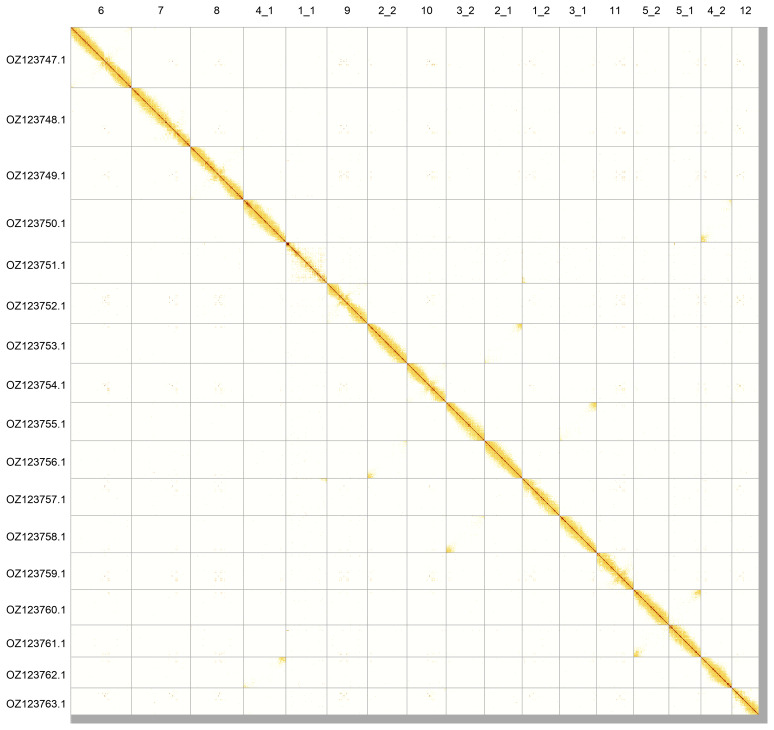
Genome assembly of *Triturus cristatus*: Hi-C contact map of the aTriCri1.1 assembly, produced in PretextView. Chromosomes are shown in order of size from left to right and top to bottom.

Most of the assembly sequence (98.78%) was assigned to 12 chromosomal-level scaffolds, with each of chromosomes 1 to 5 split into two parts for submission to INSDC databases. These chromosome-level scaffolds, confirmed by Hi-C data, are named according to size ( Figure 5; Table 3). During assembly curation, we noted that one arm of chromosome 1 (chromosome 1_1) has half-coverage, which is a completely heterozygous region related to the chromosome 1 heteromorphy of *T. cristatus* ( Macgregor & Horner, 1980; Wielstra, 2020).

**Table 3.** Chromosomal pseudomolecules in the genome assembly of *Triturus cristatus*, aTriCri1.

INSDC accession	Name	Length (Mb)	GC%
OZ123751.1	1_1	1,317.4	48
OZ123757.1	1_2	1,199.17	45.5
OZ123756.1	2_1	1,201.42	45.5
OZ123753.1	2_2	1,269.97	45
OZ123758.1	3_1	1,184.55	45.5
OZ123755.1	3_2	1,238.91	45.5
OZ123750.1	4_1	1,361.38	45.5
OZ123762.1	4_2	993.38	45.5
OZ123761.1	5_1	1,024.25	45
OZ123760.1	5_2	1,134.71	45
OZ123747.1	6	1,950.67	45
OZ123748.1	7	1,882.94	45.5
OZ123749.1	8	1,702.34	45
OZ123752.1	9	1,293.89	45.5
OZ123754.1	10	1,248.77	45
OZ123759.1	11	1,183.99	45.5
OZ123763.1	12	864.23	45.5
OZ123764.1	MT	0.02	41.5

The mitochondrial genome was also assembled. This sequence is included as a contig in the multifasta file of the genome submission and as a standalone record.

### Assembly quality metrics

The estimated Quality Value (QV) and *k*-mer completeness metrics, along with BUSCO completeness scores, were calculated for each haplotype and the combined assembly. The QV reflects the base-level accuracy of the assembly, while *k*-mer completeness indicates the proportion of expected *k*-mers identified in the assembly. BUSCO scores provide a measure of completeness based on benchmarking universal single-copy orthologues.

The combined primary and alternate assemblies achieve an estimated QV of 62.4. The *k*-mer recovery for the primary haplotype is 94.04%, and for the alternate haplotype 45.11%; the combined primary and alternate assemblies have a *k*-mer recovery of 99.07%. BUSCO v.5.7.1 analysis using the tetrapoda_odb10 reference set ( *n* = 5,310) identified 92.7% of the expected gene set (single = 88.8%, duplicated = 3.8%).

Table 2 provides assembly metric benchmarks adapted from Rhie *et al.* (2021) and the Earth BioGenome Project Report on Assembly Standards September 2024. The assembly achieves the EBP reference standard of **6.C.Q65**.

## Methods

### Sample acquisition

The genome was sequenced from one female *T. cristatus* (specimen ID NHMUK014561658, ToLID aTriCri1; Figure 1A, B, C) collected by net from a garden pond at Gilbert White’s House and Gardens in Selbourne, England on 14 August 2022 (latitude 51.0975, longitude –0.9443; Figure 1D, E) at 13:45 hrs. The individual had a snout-vent length of 82.6 mm and a mass of 12.2 g. The specimen was collected and identified by Jeff Streicher and Stephanie Holt (Natural History Museum) and preserved dry freezing (–80 °C). The voucher specimen was deposited at the Natural History Museum, London (NHMUK 2022.7580, Field ID, JWS 916).

Metadata collection for samples adhered to the Darwin Tree of Life project standards described by Lawniczak *et al.* (2022).

### Nucleic acid extraction

The workflow for high molecular weight (HMW) DNA extraction at the Wellcome Sanger Institute (WSI) Tree of Life Core Laboratory includes a sequence of procedures: sample preparation and homogenisation, DNA extraction, fragmentation and purification. Detailed protocols are available on protocols.io ( Denton *et al.*, 2023b). The aTriCri1 sample was prepared for DNA extraction by weighing and dissecting it on dry ice ( Jay *et al.*, 2023). Tissue from the liver was homogenised using a PowerMasher II tissue disruptor ( Denton *et al.*, 2023a). HMW DNA was extracted using the MagAttract v2 protocol ( Oatley *et al.*, 2023a). DNA was sheared into an average fragment size of 12–20 kb in a Megaruptor 3 system ( Bates *et al.*, 2023). Sheared DNA was purified by solid-phase reversible immobilisation, using AMPure PB beads to eliminate shorter fragments and concentrate the DNA ( Nowoshilow *et al.*, 2018; Oatley *et al.*, 2023b). The concentration of the sheared and purified DNA was assessed using a Nanodrop spectrophotometer and Qubit Fluorometer using the Qubit dsDNA High Sensitivity Assay kit. Fragment size distribution was evaluated by running the sample on the FemtoPulse system.

RNA was extracted from ovary tissue of aTriCri1 in the Tree of Life Laboratory at the WSI using the RNA Extraction: Automated MagMax™ *mir*Vana protocol ( do Amaral *et al.*, 2023). The RNA concentration was assessed using a Nanodrop spectrophotometer and a Qubit Fluorometer using the Qubit RNA Broad-Range Assay kit. Analysis of the integrity of the RNA was done using the Agilent RNA 6000 Pico Kit and Eukaryotic Total RNA assay.

### Hi-C sample preparation and crosslinking

Hi-C data were generated from the muscle of the aTriCri1 sample using the Arima-HiC v2 kit (Arima Genomics) with 20–50 mg of frozen tissue (stored at –80 °C). As per manufacturer’s instructions, tissue was fixed, and the DNA crosslinked using a TC buffer with 22% formaldehyde concentration, and a final formaldehyde concentration of 2%. The tissue was then homogenised using the Diagnocine Power Masher-II. The crosslinked DNA was digested using a restriction enzyme master mix, then biotinylated and ligated. A clean up was performed with SPRIselect beads prior to library preparation. DNA concentration was quantified using the Qubit Fluorometer v4.0 (Thermo Fisher Scientific) and Qubit HS Assay Kit, and sample biotinylation percentage was estimated using the Arima-HiC v2 QC beads.

### Library preparation and sequencing

Library preparation and sequencing were performed at the WSI Scientific Operations core.

***PacBio HiFi***

At a minimum, samples were required to have an average fragment size exceeding 8 kb and a total mass over 400 ng to proceed to the low-input SMRTbell Prep Kit 3.0 protocol (Pacific Biosciences), depending on genome size and sequencing depth required. Libraries were prepared using the SMRTbell Prep Kit 3.0 as per the manufacturer's instructions. The kit includes the reagents required for end repair/A-tailing, adapter ligation, post-ligation SMRTbell bead cleanup, and nuclease treatment. Size-selection and clean-up were carried out using diluted AMPure PB beads (Pacific Biosciences). DNA concentration was quantified using the Qubit Fluorometer v4.0 (ThermoFisher Scientific) with Qubit 1X dsDNA HS assay kit and the final library fragment size analysis was carried out using the Agilent Femto Pulse Automated Pulsed Field CE Instrument (Agilent Technologies) and the gDNA 55kb BAC analysis kit.

Samples were sequenced on a Revio instrument (Pacific Biosciences, California, USA). Prepared libraries were normalised to 2 nM, and 15 μL was used for making complexes. Primers were annealed and polymerases were bound to create circularised complexes according to manufacturer’s instructions. The complexes were purified with the 1.2X clean up with SMRTbell beads. The purified complexes were then diluted to the Revio loading concentration (in the range 200–300 pM), and spiked with a Revio sequencing internal control. Samples were sequenced on Revio 25M SMRT cells (Pacific Biosciences, California, USA). The SMRT link software, a PacBio web-based end-to-end workflow manager, was used to set-up and monitor the run, as well as perform primary and secondary analysis of the data upon completion.

***Hi-C***

For Hi-C library preparation, the biotinylated DNA constructs were fragmented using a Covaris E220 sonicator and size-selected to 400–600 bp using SPRISelect beads. DNA was then enriched using Arima-HiC v2 Enrichment beads. The NEBNext Ultra II DNA Library Prep Kit (New England Biolabs) was used for end repair, A-tailing, and adapter ligation, following a modified protocol in which library preparation is carried out while the DNA remains bound to the enrichment beads. PCR amplification was performed using KAPA HiFi HotStart mix and custom dual-indexed adapters (Integrated DNA Technologies) in a 96-well plate format. Depending on sample concentration and biotinylation percentage determined at the crosslinking stage, samples were amplified for 10–16 PCR cycles. Post-PCR clean-up was carried out using SPRISelect beads. The libraries were quantified using the Accuclear Ultra High Sensitivity dsDNA Standards Assay kit (Biotium) and normalised to 10 ng/μL before sequencing. Hi-C sequencing was performed on the Illumina NovaSeq 6000 instrument with 150 bp paired-end reads.

***RNA***

Poly(A) RNA-Seq libraries were constructed using the NEB Ultra II RNA Library Prep kit, following the manufacturer’s instructions. RNA sequencing was performed on the Illumina NovaSeq X instrument.

### Genome assembly, curation and evaluation

***Assembly***

Prior to assembly of the PacBio HiFi reads, a database of *k*-mer counts ( *k* = 31) was generated from the filtered reads using FastK. GenomeScope2 ( Ranallo-Benavidez *et al.*, 2020) was used to analyse the *k*-mer frequency distributions, providing estimates of genome size, heterozygosity, and repeat content.

The HiFi reads were first assembled using Hifiasm ( Cheng *et al.*, 2021) with the --primary option. Haplotypic duplications were identified and removed using purge_dups ( Guan *et al.*, 2020). The Hi-C reads ( Rao *et al.*, 2014) were mapped to the primary contigs using bwa-mem2 ( Vasimuddin *et al.*, 2019), and the contigs were scaffolded using YaHS ( Zhou *et al.*, 2023) using the --break option for handling potential misassemblies. The scaffolded assemblies were evaluated using Gfastats ( Formenti *et al.*, 2022), BUSCO ( Manni *et al.*, 2021) and MERQURY.FK ( Rhie *et al.*, 2020).

The mitochondrial genome was assembled using MitoHiFi ( Uliano-Silva *et al.*, 2023), which runs MitoFinder ( Allio *et al.*, 2020) and uses these annotations to select the final mitochondrial contig and to ensure the general quality of the sequence.

***Assembly curation***

The assembly was decontaminated using the Assembly Screen for Cobionts and Contaminants (ASCC) pipeline. Flat files and maps used in curation were generated via the TreeVal pipeline ( Pointon *et al.*, 2023). Manual curation was conducted primarily in PretextView ( Harry, 2022) and HiGlass ( Kerpedjiev *et al.*, 2018), with additional insights provided by JBrowse2 ( Diesh *et al.*, 2023). Scaffolds were visually inspected and corrected as described by Howe *et al.* (2021). Any identified contamination, missed joins, and mis-joins were amended, and duplicate sequences were tagged and removed. The curation process is documented at https://gitlab.com/wtsi-grit/rapid-curation.

***Assembly quality assessment***

The Merqury.FK tool ( Rhie *et al.*, 2020), run in a Singularity container ( Kurtzer *et al.*, 2017), was used to evaluate *k*-mer completeness and assembly quality for the primary and alternate haplotypes using the *k*-mer databases ( *k* = 31) computed prior to genome assembly. The analysis outputs included assembly QV scores and completeness statistics.

The genome was analysed in the blobtoolkit pipeline, a Nextflow ( Di Tommaso *et al.*, 2017) port of the previous Snakemake Blobtoolkit pipeline ( Challis *et al.*, 2020). It aligns the PacBio reads in SAMtools ( Danecek *et al.*, 2021) and minimap2 ( Li, 2018) and generates coverage tracks for regions of fixed size. In parallel, it queries the GoaT database ( Challis *et al.*, 2023) to identify all matching BUSCO lineages to run BUSCO ( Manni *et al.*, 2021). For the three domain-level BUSCO lineages, the pipeline aligns the BUSCO genes to the UniProt Reference Proteomes database ( Bateman *et al.*, 2023) with DIAMOND blastp ( Buchfink *et al.*, 2021). The genome is also divided into chunks according to the density of the BUSCO genes from the closest taxonomic lineage, and each chunk is aligned to the UniProt Reference Proteomes database using DIAMOND blastx. Genome sequences without a hit are chunked using seqtk and aligned to the NT database with blastn ( Altschul *et al.*, 1990). The blobtools suite combines all these outputs into a blobdir for visualisation.

The blobtoolkit pipeline was developed using nf-core tooling ( Ewels *et al.*, 2020) and MultiQC ( Ewels *et al.*, 2016), relying on the Conda package manager, the Bioconda initiative ( Grüning *et al.*, 2018), the Biocontainers infrastructure ( da Veiga Leprevost *et al.*, 2017), as well as the Docker ( Merkel, 2014) and Singularity ( Kurtzer *et al.*, 2017) containerisation solutions.

Table 4 contains a list of relevant software tool versions and sources.

**Table 4.** Software tools: versions and sources.

Software tool	Version	Source
BLAST	2.14.0	ftp://ftp.ncbi.nlm.nih.gov/blast/executables/blast+/
BlobToolKit	4.4.4	https://github.com/blobtoolkit/blobtoolkit
BUSCO	5.7.1	https://gitlab.com/ezlab/busco
bwa-mem2	2.2.1	https://github.com/bwa-mem2/bwa-mem2
DIAMOND	2.1.8	https://github.com/bbuchfink/diamond
fasta_windows	0.2.4	https://github.com/tolkit/fasta_windows
FastK	666652151335353eef2fcd58880bcef5bc2928e1	https://github.com/thegenemyers/FASTK
GenomeScope2.0	2.0.1	https://github.com/tbenavi1/genomescope2.0
Gfastats	1.3.6	https://github.com/vgl-hub/gfastats
GoaT CLI	0.2.5	https://github.com/genomehubs/goat-cli
Hifiasm	0.19.8-r603	https://github.com/chhylp123/hifiasm
HiGlass	44086069ee7d4d3f6f3f0012569789ec138f42b84 aa44357826c0b6753eb28de	https://github.com/higlass/higlass
MerquryFK	d00d98157618f4e8d1a9190026b19b471055b22e	https://github.com/thegenemyers/MERQURY.FK
MitoHiFi	3	https://github.com/marcelauliano/MitoHiFi
MultiQC	1.14, 1.17, and 1.18	https://github.com/MultiQC/MultiQC
Nextflow	24.04.2	https://github.com/nextflow-io/nextflow
PretextView	0.2.5	https://github.com/sanger-tol/PretextView
samtools	1.21	https://github.com/samtools/samtools
sanger-tol/ascc	-	https://github.com/sanger-tol/ascc
sanger-tol/blobtoolkit	v0.7.0	https://github.com/sanger-tol/blobtoolkit
Seqtk	1.3	https://github.com/lh3/seqtk
Singularity	3.9.0	https://github.com/sylabs/singularity
TreeVal	1.2.0	https://github.com/sanger-tol/treeval
YaHS	1.2a.2	https://github.com/c-zhou/yahs

### Wellcome Sanger Institute – Legal and Governance

The materials that have contributed to this genome note have been supplied by a Darwin Tree of Life Partner. The submission of materials by a Darwin Tree of Life Partner is subject to the **‘Darwin Tree of Life Project Sampling Code of Practice’**, which can be found in full on the Darwin Tree of Life website here. By agreeing with and signing up to the Sampling Code of Practice, the Darwin Tree of Life Partner agrees they will meet the legal and ethical requirements and standards set out within this document in respect of all samples acquired for, and supplied to, the Darwin Tree of Life Project.

Further, the Wellcome Sanger Institute employs a process whereby due diligence is carried out proportionate to the nature of the materials themselves, and the circumstances under which they have been/are to be collected and provided for use. The purpose of this is to address and mitigate any potential legal and/or ethical implications of receipt and use of the materials as part of the research project, and to ensure that in doing so we align with best practice wherever possible. The overarching areas of consideration are:

•   Ethical review of provenance and sourcing of the material

•   Legality of collection, transfer and use (national and international)

Each transfer of samples is further undertaken according to a Research Collaboration Agreement or Material Transfer Agreement entered into by the Darwin Tree of Life Partner, Genome Research Limited (operating as the Wellcome Sanger Institute), and in some circumstances other Darwin Tree of Life collaborators.

## Data Availability

European Nucleotide Archive: Triturus cristatus (warty newt). Accession number PRJEB75439; https://identifiers.org/ena.embl/PRJEB75439. The genome sequence is released openly for reuse. The *Triturus cristatus* genome sequencing initiative is part of the Darwin Tree of Life Project (PRJEB40665), the Sanger Institute Tree of Life Programme (PRJEB43745) and the Vertebrate Genomes Project (PRJNA489243). All raw sequence data and the assembly have been deposited in INSDC databases. The genome will be annotated using available RNA-Seq data and presented through the Ensembl pipeline at the European Bioinformatics Institute. Raw data and assembly accession identifiers are reported in Table 1 and Table 2.

## Author information

Members of the Natural History Museum Genome Acquisition Lab are listed here: https://doi.org/10.5281/zenodo.12159242.

Members of the Darwin Tree of Life Barcoding collective are listed here: https://doi.org/10.5281/zenodo.12158331.

Members of the Wellcome Sanger Institute Tree of Life Management, Samples and Laboratory team are listed here: https://doi.org/10.5281/zenodo.12162482.

Members of Wellcome Sanger Institute Scientific Operations: Sequencing Operations are listed here: https://doi.org/10.5281/zenodo.12165051.

Members of the Wellcome Sanger Institute Tree of Life Core Informatics team are listed here: https://doi.org/10.5281/zenodo.12160324.

Members of the Tree of Life Core Informatics collective are listed here: https://doi.org/10.5281/zenodo.12205391.

Members of the Darwin Tree of Life Consortium are listed here: https://doi.org/10.5281/zenodo.4783558.
