# Supplementary material for: The genome sequence of the great crested newt, Triturus cristatus (Laurenti, 1768)
Source: Wellcome Open Res. 2025 May 23;10:270. [Version 1] doi: 10.12688/wellcomeopenres.24257.1 (PMC12134737; doi:10.12688/wellcomeopenres.24257.1)
Supplement: Peer review history

# Reviewer report: Reviewer response for version 1

**DOI:** 10.21956/wellcomeopenres.26754.r124336

**Published:** 2025-06-03

**Authors:** Andrew J Crawford

**Affiliations:** 1 Universidad de Los Andes Bogotá, Bogotá, Bogotá, Colombia

 Summary:

 This genome assembly report is remarkable in the level of detail and links to further resources, maximizing transparency and replicability of this work. The result is a massive genome assembled to remarkably high standards. The final assembly has a total length of 22,324.62 Mb in 1,303 scaffolds, with 4,698 gaps, and a scaffold N50 of 1248.77 Mb.

 Minor comments:

Abstract:

 Common name of amphibians are usually written with frist letters in upper case (see AMNH Amph Spp World, AmphibiaWeb, and your keywords for examples). 

 "22,324.62 megabases" could be more clear written as "22.3 Gb" here and throughout, but that’s a personal opinion. 

Background:

 Paragraphs 1 and 3: see comment above on capitalizing common name. 

 Line 9: slight misspelling of "Batrachochytrium salamandrivorans". 

 Paragraph 3, line 1: I think the ';' should be a ':'.

 Sentence 2: 'each variant' of how many?  Just 2 variants or many are found in the population?

 Paragraph 4: I actually prefer the ':' between higher taxonomic names, as you have here, although in Abstract & Taxonomy sections the authors used the ';' (as does NCBI). 

 I believe 'C-value' is more typically written with an uppercase 'C'.  Perhaps also provide for the reader a conversion of pg to Gb? 

 There is also the 2019 *A. mexicanum *assembly by JJ Smith et al., and new *Ambystoma* assembly weighing in at 29 Gb https://www.ncbi.nlm.nih.gov/datasets/genome/GCF_040938575.1/

 ‘Sanger Tree of life Project ’, capitalize the ‘L’. Regarding ‘Vertebrate Tree of Life Project’, do you perhaps mean the “Vertebrate Genome Project”?

‘Sequencing data’ section:

 ‘36 coverage’, missing an ‘X’ or ‘-fold’?

 Thanks for including RNAseq data. https://www.ncbi.nlm.nih.gov/sra/ERR13493948  That’s very helpful.  The community looks forward to the subsequent annotation.

 Results state “misjoins or missing joins” and the Methods say “missed joins and mis-joins”. Perhaps homogenize the terminology?

Methods:

 Line 1. Your specimen ID “NHMUK014561658” gets no hits at the Museum, https://data.nhm.ac.uk/search , but these 2 work: “NHMUK 014561658” or “NHMUK:014561658”.  Just to be kind, perhaps add the ‘:’ in there. Ah, I see on Line 5 you have a different code “NHMUK 2022.7580” which also works.  Just curious, why a ‘specimen ID’ and a museum voucher number? 

 While I absolutely support this statement, “Metadata collection for samples adhered to the Darwin Tree of Life project standards described by Lawniczak et al. (2022),” having this phrase comprising by itself a whole new paragraph is actually not clear to me.  Does this mean the previous paragraph followed Lawniczak et al., or does this mean the metadata online follows these recommendations? Perhaps you could state what metadata provided and then cite this reference?  Just a thought.

 Denton et al. (2023b) now has a version 2 you could cite directly: https://www.protocols.io/view/sanger-tree-of-life-wet-laboratory-protocol-collec-8epv5xxy6g1b/v2

*PacBio HiFi*

 Add space in ‘55kb’.

 Pacific Biosciences is cited 4 times, half the time with the localtion, half without. Most other citations to manufactures do not include their location. And some products had no citation to the manufacture. Perhaps be more consistent? 

 ‘analyses’ plural.

“Wellcome Sanger Institute – Legal and Governance”

 Line 4. Just curious, the whole animal went into -80°C, or it was ethanized first and the tissues went into -80°C?  So, tissues were later taken out of a rock-solid frozen newt?   (No liquid nitrogen was involved?)

 I am not seeing an ethics statement concerning Institutional Animal Care & Use approval.  The section ‘Wellcome Sanger Institute – Legal and Governance’ points me to a document called ‘Sampling Code of Practice’ here https://www.darwintreeoflife.org/project-resources/ which offers me a PDF here https://www.darwintreeoflife.org/wp-content/uploads/2023/10/Template-DToL-Sampling-Code-Of-Practice.pdf which has an Appendix that mentions “Sampling methods for relevant animals must be humane and consistent with current legal requirements and good practice.” I admit I am unfamililar with UK or English regulations around animal care and use, but in many countries one is expected to provide evidence of approval for euthanasia methods by an institutional committee, such as an IACUC in the USA.  I would recommend providing the reader with some assurance that freezing a live newt in -80°C freezer is an approved method.  Alternatively, if the animal was anestitized first, one ought to state the method and provide evidence of committee approval and/or a current reference to veterinary recommendations for the chosen method of euthanasia. 

Are sufficient details of methods and materials provided to allow replication by others?

Yes

Is the rationale for creating the dataset(s) clearly described?

Yes

Are the datasets clearly presented in a useable and accessible format?

Yes

Are the protocols appropriate and is the work technically sound?

Yes

Reviewer Expertise:

Evolutionary biology and genomics of amphibians.

I confirm that I have read this submission and believe that I have an appropriate level of expertise to confirm that it is of an acceptable scientific standard.

# Reviewer report: Reviewer response for version 1

**DOI:** 10.21956/wellcomeopenres.26754.r124338

**Published:** 2025-05-29

**Authors:** R Alexander Pyron

**Affiliations:** 1 The George Washington University Museum and Textile Museum, Washington, District of Columbia, USA

The Data Note by Streicher et al. describes one of only ~5 chromosome-scale salamander genomes, an important early achievement in urodelan genomics. I only have a few minor suggestions.

 The section on the collection locality in “Background” – second paragraph – is very interesting and important natural-history and actual history. However, it feels a bit out of place, going from the newt in the first paragraph, to Gilbert White in the second, back to newts in the third. I would suggest moving it either to the Methods, or to the end of the Background.

 In Genome sequence Report, Sequencing data, the sentence “Based on the estimated genome size, the sequencing data provided approximately 36 coverage” I believe should be “36x coverage.”

 The authors do not seem to explain why they did not use the Hi-C data with the Hi-C mode of hifiasm to produce phased/haplotype-resolved assembles for aTriCri1. Have they explored this?

 The authors report sequencing RNA, but they don’t report anything about the quality or any attempted assemblies. It seems like even a basic de novo assembly in Trinity or similar, with BUSCO/compleasm scores, would be a good complement to the main genome. Also, was the sequencing strand-specific, for downstream analysis?

 Perhaps this will be saved for a later manuscript or considered beyond the scope of a Data Note, but the authors do not really provide much context for how this genome compares to the other existing high-quality salamander assemblies such as Ambystoma mexicanum and Pleurodeles waltl.

Are sufficient details of methods and materials provided to allow replication by others?

Yes

Is the rationale for creating the dataset(s) clearly described?

Yes

Are the datasets clearly presented in a useable and accessible format?

Yes

Are the protocols appropriate and is the work technically sound?

Yes

Reviewer Expertise:

Evolutionary genomics in salamanders

I confirm that I have read this submission and believe that I have an appropriate level of expertise to confirm that it is of an acceptable scientific standard.
